# Sexually dimorphic and asymmetric effects of embryonic ethanol exposure on hypocretin/orexin neurons as related to behavioral changes in zebrafish

**DOI:** 10.1038/s41598-021-95707-y

**Published:** 2021-08-09

**Authors:** Adam D. Collier, Nushrat Yasmin, Nailya Khalizova, Samantha Campbell, Amanda Onoichenco, Milisia Fam, Avi S. Albeg, Sarah F. Leibowitz

**Affiliations:** Laboratory of Behavioral Neurobiology, The Rockefeller University, 1230 York Avenue, New York, NY 10065 USA

**Keywords:** Developmental biology, Neuroscience, Development of the nervous system, Diseases of the nervous system, Feeding behaviour, Neurogenesis, Reward, Addiction

## Abstract

Neurons expressing the neuropeptide hypocretin/orexin (Hcrt) in the hypothalamus promote reward-related behaviors including alcohol consumption and are shown in rodents and zebrafish to be stimulated by embryonic exposure to ethanol (EtOH). We used here in zebrafish three-dimensional analyses of the entire population of Hcrt neurons to examine how embryonic EtOH exposure at low-moderate concentrations (0.1% or 0.5% v/v) alters these neurons in relation to behavior. We found that EtOH in the water for 2 h (22–24 h post fertilization) increases the number of Hcrt neurons on the left but not right side of the brain through a stimulation of cell proliferation, this is accompanied by a decrease in locomotor activity under novel conditions but not after habituation, and these effects are evident in both larvae and adults indicating they are long lasting. Our analyses in adults revealed sexually dimorphic effects, with females consuming more EtOH-gelatin and exhibiting more freezing behavior along with an asymmetric increase in Hcrt neurons and males exhibiting increased aggression with no change in Hcrt. These findings suggest that a long lasting, asymmetric increase in Hcrt neurons induced by EtOH results from an asymmetric increase in proliferation specific to Hcrt and contributes to behavioral changes in females.

## Introduction

With about 1 in 9 women reporting alcohol use during pregnancy^1^, clinical evidence has accumulated showing that prenatal exposure to ethanol (EtOH) has detrimental, long-term effects on the health of offspring^2–4^. Even low levels of EtOH produce behavioral dysfunction including increased generalized alcohol use^5,6^ and alcohol consumption during adolescence^7,8^, suggesting that no amount of alcohol during pregnancy is safe^1^. This is supported by rodent studies in our laboratory^9^ and others^10^ showing prenatal alcohol exposure at low-to-moderate doses to increase alcohol consumption. It is also evident in our studies of zebrafish^11^, a species that has high physiological and genetic homology to humans, a comparable CNS that develops early and rapidly^12^, and a sophisticated behavioral repertoire including voluntary consumption of EtOH-gelatin that produces pharmacologically relevant blood EtOH concentrations^13^. Notably, embryonic exposure of zebrafish to EtOH in the water at low-moderate doses has similar behavioral effects^14^ to those produced by prenatal exposure to EtOH in rodents^15^ and humans^7^. Moreover, these behavioral effects in zebrafish as well as rats are accompanied by an increase in number of developing neurons in the hypothalamus^16^, specifically neurons that express orexigenic peptides known to promote alcohol consumption^11,13,17,18^, and also an increase in radial glia progenitor cells and microglia in the hypothalamus^19^, indicating that EtOH has stimulatory effects on diverse cell types.

In the present study, we focus on the neuropeptide hypocretin/orexin (Hcrt), which is expressed solely in neuronal populations within the hypothalamus. These Hcrt neurons are of particular interest because with widespread projections throughout the brain, they have an important role in reward processing^20^ and in mediating related behaviors such as arousal and anxiety^21^. The number of Hcrt neurons at baseline correlates positively in rats with motivation for cocaine and is higher in humans addicted to heroin^22,23^. In addition, the role of Hcrt in driving reward related behaviors is supported by evidence showing Hcrt neuron number and activity to be upregulated by substance abuse^24–26^. Further, genetic knockdown of Hcrt neurons reduces motivation for cocaine, and pharmacological blockade of Hcrt receptors suppresses motivation for and intake of fentanyl and palatable food^22,25–26^. Notably, the role of Hcrt in mediating specifically alcohol related behaviors is demonstrated by studies showing that pharmacological antagonism of Hcrt receptors reduces ethanol self-administration and reinstatement in alcohol-preferring rats^27^, and also reduces binge-like consumption of EtOH, but not sucrose intake, in mice^28^.

Taking advantage of the attributes of zebrafish, such as their optical transparency permitting three-dimensional analyses of the total population of Hcrt neurons, we sought here to characterize changes in the Hcrt system induced by EtOH at low-moderate doses that are physiologically relevant to humans prenatally exposed to EtOH. Exposure to EtOH at the low-moderate doses of 0.1% and 0.5% (v/v) used in the current study are expected to result in approximate blood ethanol concentrations (BEC) of 0.024 and 0.115 g/dl^29^, respectively, which are comparable to BEC (0.005 to 0.212 g/dl) detected at birth in human neonates prenatally exposed to alcohol^30^. There is evidence in zebrafish^11,31^ and rats^17,32^ that EtOH at low concentrations can stimulate both cell proliferation and neurogenesis, with this effect occurring in Hcrt neurons as suggested by a study of hypothalamic sections in rats^17^. Asymmetric effects of EtOH on Hcrt neurogenesis may also be important in light of our earlier finding that the EtOH-induced increase in number of Hcrt neurons is stronger on the left than right side^31^, and of evidence in other studies showing asymmetry of neuronal development to be an important factor that contributes to behavioral disturbances and the development of alcohol use disorder^33^.

With little information on the similarities of EtOH’s effects on zebrafish behavior to behavioral effects widely characterized in rodents, we recently established a quantitative assay for measuring voluntary EtOH intake in zebrafish, using the number of 10% ethanol-gelatin block bites, and found this measure to be positively correlated with and predictive of BEC in the consuming adult zebrafish^34^. Additionally, while evidence suggests that early EtOH exposure in zebrafish and rats increases alcohol consumption later in life^9,11^, studies of alcohol-related behaviors such as locomotor activity and anxiety^11,35^ have yielded mixed results, with these behaviors both stimulated^11^ and suppressed^35,36^ by EtOH. These differences may be due to differences in environmental conditions of the test, occurring under novel conditions or after habituation to the environment, with Hcrt possibly involved as there is evidence suggesting that Hcrt neurons have a greater function in mediating behavioral responses under novel conditions than they have in familiar environmental contexts^37^.

Prior neurobehavioral studies suggest that the number of Hcrt neurons changes naturally throughout development^38^, along with changes in locomotor and anxiety-like behaviors^39^. There are only a few reports in any species examining both the brain and behavior at multiple stages of development^11,13^, and no studies of Hcrt neurons in adult subjects after embryonic EtOH exposure, only in young adolescent rats^35^ and zebrafish^11^. With sex being a critically important biological variable for understanding the role of neuronal systems in controlling behavior and zebrafish rarely studied for their sex differences, we also expanded our analysis of adult subjects to include a direct comparison of female and male fish to determine if the stimulatory effects of embryonic EtOH exposure on Hcrt neurons and different behaviors are sexually dimorphic, perhaps stronger in females as shown in rats^35,40^.

Thus, this investigation of embryonic EtOH’s effects at low-moderate concentrations in zebrafish, on the total population of Hcrt neurons and on behaviors at different ages, will allow us to determine if the increase in Hcrt neuronal number with asymmetric properties is related to changes in: (1) hypothalamic cell proliferation and specifically of Hcrt neurons; (2) behaviors that occur under novel conditions but not after habituation and early in development as well as in adulthood; and (3) adult behaviors including voluntary EtOH consumption and aggression. It will also allow us to determine if these neuronal and behavioral effects are sexually dimorphic in adults. Our results provide evidence in zebrafish that embryonic EtOH asymmetrically increases the proliferation and number of Hcrt neurons, produces behavioral changes specifically under novel conditions, increases voluntary EtOH consumption and aggressive behaviors in adults, and shows these neuronal and behavioral effects to be long-lasting and strongly sex dependent as suggested in the rat.

## Materials and methods

### Animals and housing

Transgenic *hcrt:EGFP*^41^ zebrafish (*Danio rerio*) derived from a wildtype AB strain were used for this study. Adult zebrafish were group housed in 3 L tanks (Aquatic Habitat, Apopka, FL), bred, and their embryos raised within an AAALAC accredited facility as described previously^16,34^. All protocols were approved by the Rockefeller University Institutional Animal Care and Use Committee and followed the NIH Guide for the Care and Use of Laboratory Animals. The study was carried out in compliance with the ARRIVE guidelines. Zebrafish were sexed by an experienced researcher by assessing multiple parameters, such as body shape and coloration, as has been previously reported^42,43^.

### Embryonic EtOH treatment

All *hcrt:EGFP* embryos were divided into three conditions, 0.0% (control), 0.1% EtOH or 0.5% EtOH (v/v). At 22 h post-fertilization (hpf), the embryos were placed in a fresh solution of their respective conditions, and then returned to the incubator for a 2-h exposure period. After this, the 24 hpf embryos were washed 3 × in embryo media and returned to the incubator.

### EdU labeling

Larval cell proliferation labeling was achieved with the Click iT EdU Cell Proliferation Kit Alexa Fluor 647 (Invitrogen, Waltham, MA). 24 hpf zebrafish were transferred into a 10 mM solution of EdU with 15% dimethyl sulfoxide (DMSO) in embryo media and incubated for 30 min on ice, and then raised to 6 days post-fertilization (dpf). They were fixed overnight in ice-cold 4% PFA in phosphate buffer. Following 1X-PBS washes, brains were excised and incubated in EdU reaction mixture for 3 h at RT. Brains were then washed in 1X-PBS, blocked in 2% normal donkey serum (NDS) for 2 h prior to overnight incubation at 4 °C in primary rabbit anti-GFP antibody (Life Technologies Corp, Eugene, OR, 1:200) and were then incubated for 3 h in a solution of 4,6-Diamidino-2-phenylindole (DAPI, 1:5000) and secondary antibody Alexa 488 anti-rabbit (Abcam, Cambridge, MA, 1:400). Samples were then washed in 1X-PBS and mounted with ProLong Diamond Antifade Mountant (Thermo Fisher Scientific, Waltham, MA) and cover slipped on a glass slide prior to imaging.

### Live imaging

Live imaging samples of 6 dpf *hcrt:EGFP* larvae were mounted within 1% low-melting-point agarose containing Tricaine Methanesulfonate (15 mM) (Pentair, Cary, USA) within a 15-mm diameter well of a metal slide that had a glass coverslip adhered to the bottom, as previously reported^34,44^.

### iDISCO

iDISCO whole-brain clearing was performed as previously reported^45,46^, with modifications specific for the zebrafish brain. Adult fish were fixed overnight at 4 °C in 4% PFA in phosphate buffer followed by brain dissections and dehydration in a series of methanol dilutions for 10 min at RT. Samples were washed 3 × 10 min in dichloromethane (DCM), then 3 × 10 min in 100% methanol, bleached in H_2_O_2_/methanol (1: 5 v/v) for 2 h at 4 °C, rehydrated in a series of methanol dilutions for 10 min at RT, and washed in permeabilization solution (5% DMSO/0.3 M Glycine/PTxWH Buffer) 3 × 30 min at RT. Samples were washed in PTxWH buffer [450 ml H_2_O, 50 ml PBS 10 ×, 500 µl Triton X-100, 250 µl Tween-20, 50 µl Heparin (from 20 mg/ml stock)] 3 × 30 min at RT with shaking and returned to 4 °C. Thereafter, samples were blocked in 3% NDS/PTxWH buffer overnight at 37 °C and incubated with rabbit anti-GFP antibody (Life Technologies Corp, Eugene, OR, 1:250) in antibody buffer (3% NDS, 0.2% Triton X-100, 2% DMSO, PTxWH) for 2 days at 37 °C, followed by washes in PTxWH 6 × 1 h and soaking in PTxWH overnight. Samples were then incubated in secondary antibody Alexa 488 donkey anti-rabbit (Abcam, Cambridge, MA, 1:200) and then washed in PTxWH 6 × 1 h and soaked in PTxWH overnight. The brains were then embedded into 1% agarose blocks and dehydrated in a series of methanol dilutions for 10 min at RT and were washed with 100% DCM 3 × 20 min, cleared in 100% dibenzyl ether (DBE) 1 × 2 h at RT, and then kept in new DBE at RT in the dark until imaging.

### Microscopy and image analysis

Z-stack images of the whole anterior hypothalamus (AH) in larvae were acquired with an inverted Zeiss LSM 780 laser scanning confocal microscope with a 40 ×/1.3 objective lens with a 0.21 mm working distance and a z step of 1 μm. iDISCO samples were imaged on a light-sheet microscope (Ultramicroscope II, LaVision Biotec) using a 4 ×/0.3 objective lens equipped with a 6 mm working distance dipping cap with a z step of 0.5 μm. All images were deconvolved using AutoQuant X3 software (Media Cybernetics, Rockville, MD) and analyzed using Imaris 9.6.1 software (Bitplane, Zurich, Switzerland). The AH was cropped with the ‘Surface’ function in Imaris using dimensions reported in the zebrafish brain browser^47^. The ‘Spots’ function in Imaris was used for counts of DAPI, EdU, and Hcrt and was followed by manual correction. Instances of EdU colocalization with Hcrt neurons nuclei were manually quantified.

### Larval behavioral testing

Following the 0.0%, 0.1% or 0.5% EtOH treatment, *hcrt:EGFP* embryos at 6 dpf underwent behavioral testing over a 10-min period, with measurements recorded over min 1 and min 10 to compare the behavioral responses under novel conditions and after habituation, respectively. Zebrafish were individually moved into a well of a standard 6-well culture plate containing water from their home tank. Behavior was recorded overhead using an iPad. The videos were uploaded to BehaviorCloud, a video tracking service, and were analyzed for locomotor activity using freezing time (s), distance traveled (cm), and average velocity (cm/s) for the entire exploratory area^48^. Freezing time was determined by measuring the time animals moved less than ¼ of their total body length.

### Adult behavioral testing

The novel tank test was conducted on adult zebrafish to assess their locomotion and anxiety-like behavior. The *hcrt:EGFP* embryos in each condition (control, 0.1% EtOH, and 0.5% EtOH) were group housed in 3 l tanks until adulthood. On the day of testing, each fish was removed from their group tank, individually placed in a 1.5 l tank, and recorded for 10 min. The videos were uploaded to BehaviorCloud to measure locomotor activity, with measures of time spent freezing (s), distance traveled (cm), and average velocity (cm/s) for the entire tank. To measure anxiety-like behavior, the area of the 1.5 l tank was divided equally into a top and bottom zone, and the measures of time in top zone (% of total) and distance traveled in top zone (% of total) were calculated during min 1 and min 10^11,49^. To measure aggression, a mirror was fitted to the side of the 1.5 l tank^11^. Two areas denoting engagement with the mirror were established: a contact zone (within 0.5 cm from the mirror) and approach zone (within 2.5 cm from the contact zone), and the measures of time in contact zone (s) and approach zone (s) were calculated.

### Voluntary EtOH consumption in adult zebrafish

After the novel tank test, adult female and male fish were individually housed in 1.5 l tanks and trained to consume 10% EtOH gelatin, as we have described previously^11^. A gelatin stock was first prepared by stirring one packet (1.8 g) of gelatin (Knox Gelatin, Kraft Foods, Northfield, IL) with 120 ml of DI water on a hot plate until completely dissolved. Then, 18 ml of stock gelatin and 6 ml of either DI water or EtOH diluted in DI water were combined to form a final concentration of 0% or 10% EtOH. After being chilled at 4 °C for 10 min, fresh brine shrimp nauplii (Brine Shrimp Direct, Ogden, UT) were suspended evenly throughout the gelatin, and returned to 4 °C until set. To reduce gelatin dissolution, each tank was brought to RT an hour prior to testing. For 5 days, 2 h into the light cycle (11:00 am), each fish was given four cubes (250 mg each) of control gelatin for 10 min. Then for 4 days, fish from each EtOH condition were given either control or EtOH gelatin at 11:00 am, fed brine shrimp 2 h later at 1:00 pm, and then food restricted until the next day. On the final 2 days of feeding, the number of bites taken of EtOH-gelatin was manually scored over 10 min.

### Statistical analysis

In larval zebrafish, data from experiments evaluating Hcrt neuron number, cell proliferation, and behavior were analyzed using a two-way ANOVA, which tested the main effects of EtOH (between-subject) and brain side (within-subject) or the main effects of EtOH and time (within-subject), and the interactions between these variables. In adult zebrafish, the data for females and males were analyzed using a three-way ANOVA, which tested the main effects of either EtOH (between-subject), sex (between-subject), and gelatin-condition (between-subject), or EtOH, sex, and time (within-subject), and the interactions between these variables. Significant interactions were followed by post-hoc Holm-Sidak’s multiple comparisons test. Pearson’s correlation was used to determine the relationship between measures of cell proliferation and Hcrt neurons in the AH. Normality was assessed using Shapiro–Wilk and Kolmogorov–Smirnov tests. All tests were two-tailed, and significance was determined at *p* < 0.05, with Bonferroni adjusted alpha levels used when appropriate. All data are presented as mean ± SEM and were analyzed using Prism (version 8, GraphPad, San Diego, CA).

## Results

### Embryonic EtOH increases cellular proliferation similarly on both sides of the AH

**Figure 1:**
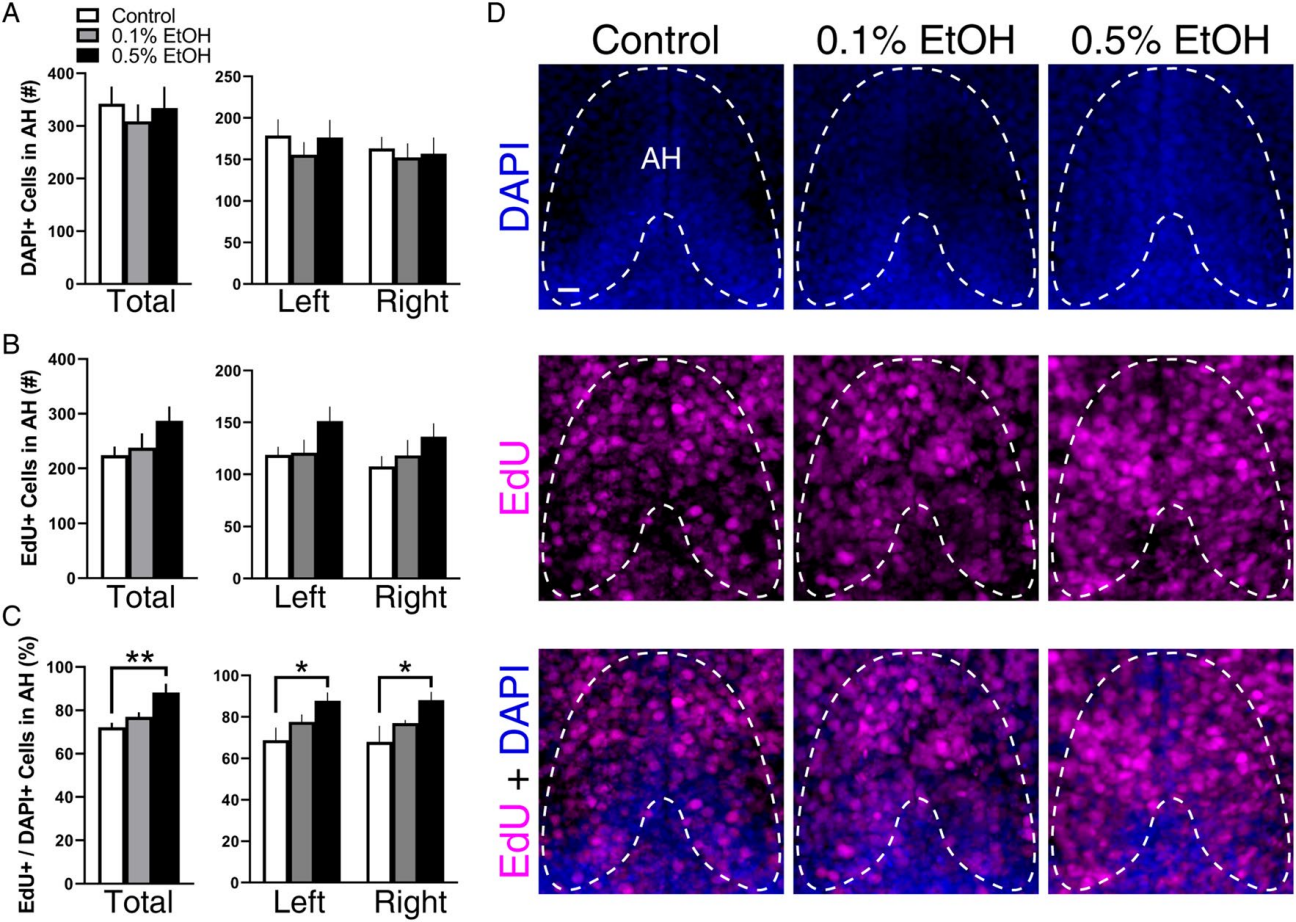
Effects of embryonic EtOH from 22 to 24 hpf on cell proliferation in the entire anterior hypothalamus (AH) measured using EdU labeling in 6 dpf larval zebrafish. (**A**) EtOH at 0.1% (n = 6) and 0.5% (n = 6) produces no change in total number and left/right DAPI-positive cells compared to control (n = 5). (**B**) EtOH at 0.1% and 0.5% has no effect on total number and left/right EdU-positive cells compared to control. (**C**) EtOH at 0.5% but not 0.1% significantly increases the total and left/right percentage of DAPI cells that are positive for EdU compared to control. (**D**) These effects of EtOH are illustrated in representative confocal images with the AH outlined. While showing no effect of 0.1% and 0.5% EtOH on single labeled DAPI-positive (blue in top row) or EdU-positive (magenta in middle row) cells compared to control, the double labeling of EdU + DAPI cells (blue and magenta overlay in bottom row) shows that 0.5% EtOH compared to control and 0.1% EtOH significantly increases the percentage of EdU-positive DAPI cells on both sides of the AH. Scale bar, 10 µm. Results are shown as means ± standard errors. *p < 0.05, **p < 0.01. *AH* anterior hypothalamus.

Our first goal was to determine, by quantifying the number of cells positive for the proliferation marker EdU, if embryonic exposure to EtOH at the low-moderate concentrations of 0.1% and 0.5% v/v from 22 to 24 hpf increases cellular proliferation throughout the AH of 6 dpf larval zebrafish. Analysis of total cell count showed that EtOH, while having no effect on the number of DAPI cells (Fig. 1A) or EdU-positive cells (Fig. 1B), significantly increased the percent of DAPI cells that were EdU positive (*F*(2, 14) = 7.501, *p* = 0.0061), specifically at the 0.5% concentration (*p* = 0.0043) (Fig. 1C). While there was no significant interaction between EtOH treatment and brain side, the ANOVA revealed a significant main effect of brain side on the number of both DAPI (*F*(1, 14) = 8.253, *p* = 0.0123) and EdU (*F*(1, 14) = 4.630, *p* = 0.0494) cells and a significant main effect of EtOH on the percent of EdU-positive DAPI cells (*F*(2, 14) = 4.961, *p* = 0.0235). Further, while revealing no differences between control and EtOH conditions for DAPI (Fig. 1A) and EdU cell number (Fig. 1B), the post-hoc tests showed that the number of EdU-positive DAPI cells was significantly increased by 0.5% EtOH on both the left (*p* = 0.0212) and right (*p* = 0.0108) sides of the brain (Fig. 1C). This indicates that the EtOH-induced increase in cell proliferation occurs symmetrically on both sides of the brain, as illustrated in the photomicrographs (Fig. 1D).

### Embryonic EtOH increases Hcrt neuron number and Hcrt proliferation asymmetrically only on the left side

**Figure 2:**
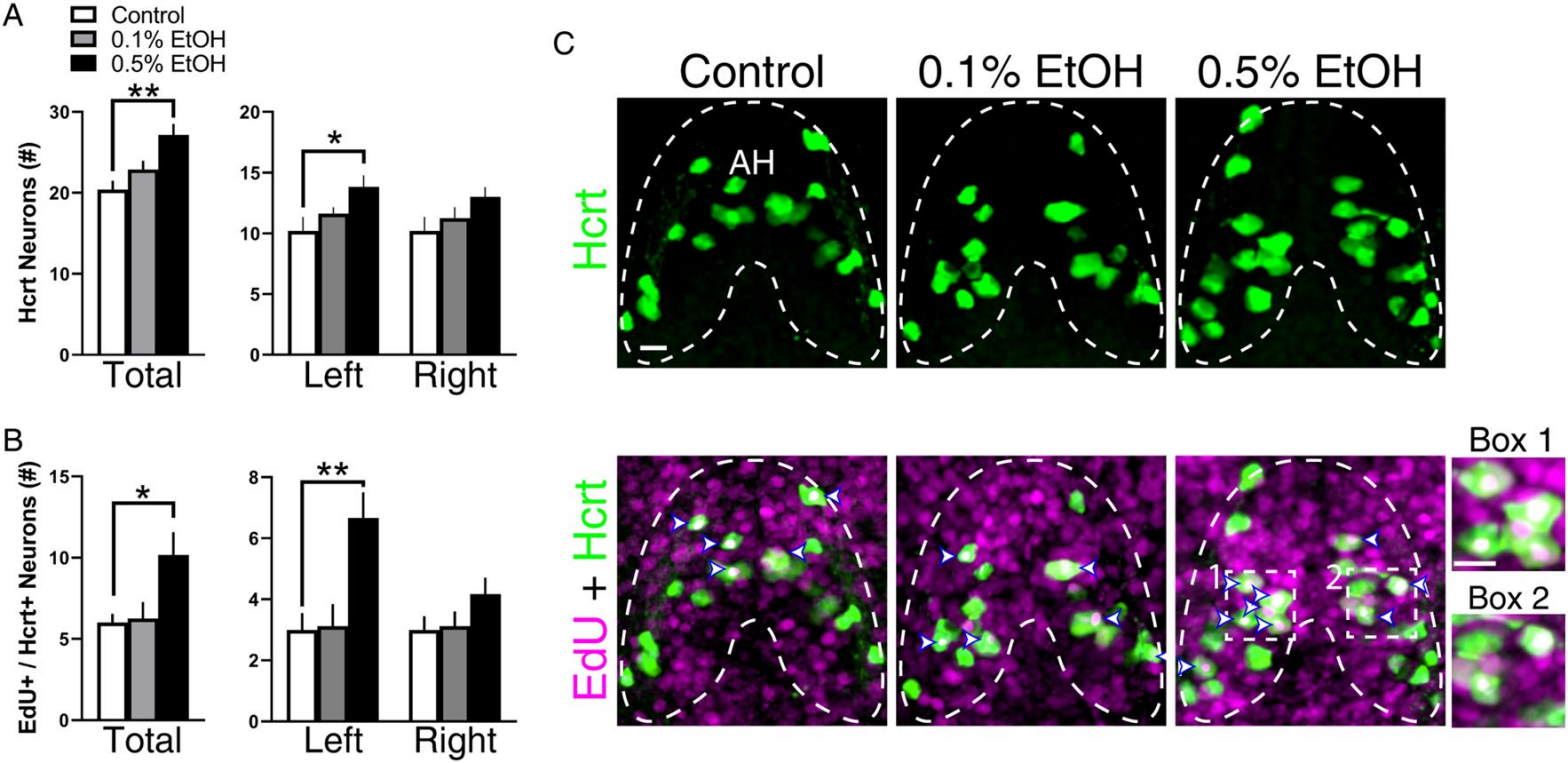
Effects of embryonic EtOH from 22 to 24 hpf on Hcrt neuron number and Hcrt proliferation in the AH measured using immunofluorescence and EdU labeling in 6 dpf larval zebrafish. (**A**) EtOH at 0.5% (n = 7) but not 0.1% (n = 8) significantly increases the total number of Hcrt neurons and the number of Hcrt neurons on the left but not right side of the brain compared to control (n = 5). (**B**) EtOH at 0.5% but not 0.1% increases the total number and left side number of Hcrt neurons labeled with EdU compared to control. (**C**) These effects are illustrated in representative confocal images with the AH outlined. They show that 0.5% but not 0.1% compared to control increases the total and left side number of GFP-positive Hcrt neurons (green in top row) and the total and left side number of EdU-labeled (magenta in bottom row) + GFP-positive Hcrt neurons (overlay, white in bottom row), with double-labeling neurons identified by white arrow heads outlined in blue with examples on the left and right enlarged within boxes 1 and 2, respectively. Scale bars, 10 µm. Results are shown as means ± standard errors. *p < 0.05, **p < 0.01. *AH* anterior hypothalamus.

We quantified the number of Hcrt neurons present in the AH of 6 dpf larvae using IF and found embryonic EtOH to increase the total number of Hcrt neurons (*F*(2,16) = 6.885, *p* = 0.0070), with this effect occurring at the 0.5% (*p* = 0.0045) but not 0.1% (*p* = 0.1572, *ns*) concentration compared to control (Fig. 2A), as shown in the photomicrographs (Fig. 2C). To determine if EtOH affected the proliferation specifically of Hcrt neurons, we measured Hcrt and EdU double-labeled neurons and found the number of Hcrt neurons that co-labeled EdU to be increased (*F*(2,17) = 5.657, *p* = 0.0131) specifically by 0.5% EtOH (*p* = 0.0251), suggesting an increase in Hcrt neurogenesis (Fig. 2B) as shown in the photomicrographs (Fig. 2C). To directly relate the increase in number of Hcrt neurons to cell proliferation in the AH, we performed Pearson’s correlations and found the total number of Hcrt neurons in the AH to be strongly, positively correlated with the number of EdU-positive Hcrt neurons (r =  + 0.751, *p* = 0.001) as well as EdU-positive DAPI cells (r =  + 0.690, *p* = 0.003), supporting the idea that the increased number of Hcrt neurons is due to an EtOH-induced increase in cell proliferation.

We next sought to determine whether the EtOH-induced increase in the number of Hcrt neurons occurs symmetrically or asymmetrically in the AH. While there was no interaction between embryonic EtOH and brain side, the ANOVA revealed a significant main effect of EtOH on the number of Hcrt neurons (*F*(2, 16) = 6.885, *p* = 0.007), and the post-hoc analysis showed 0.5% EtOH to cause a significant increase in Hcrt neuron number on the left (*p* = 0.0197) but not right (*p* = 0.1230, ns) side of the brain (Fig. 2A) as shown in the photomicrographs (Fig. 2C). Additionally, analysis of the number of EdU-positive Hcrt neurons revealed a significant interaction between embryonic EtOH and brain side (*F*(2, 17) = 4.588, *p* = 0.0255) with a main effect of EtOH (*F*(2, 17) = 5.657, *p* = 0.0131), and the post-hoc analysis showed a significant increase at 0.5% EtOH compared to control on the left (*p* = 0.0118) but not right (*p* = 0.2846, *ns*) side (Fig. 2B), as shown in the photomicrographs (Fig. 2C). Pearson’s correlations revealed significant positive correlations only on the left side of the AH, between the number of Hcrt neurons and EdU-positive DAPI cells on the left (r =  + 0.594, *p* = 0.015) but not right (r =  + 0.269, *p* = 0.296) and the number of Hcrt neurons and EdU-positive Hcrt neurons on the left (r =  + 0.615, *p* = 0.011) but not right (r =  + 0.175, *p* = 0.501). Together, these findings support the idea that the EtOH-induced, asymmetric increase in Hcrt neuron number only on the left is due to an increase in the proliferation and Hcrt neurogenesis.

### Embryonic EtOH asymmetrically increases Hcrt neurons in larval and adult zebrafish, with females more affected than males

**Figure 3:**
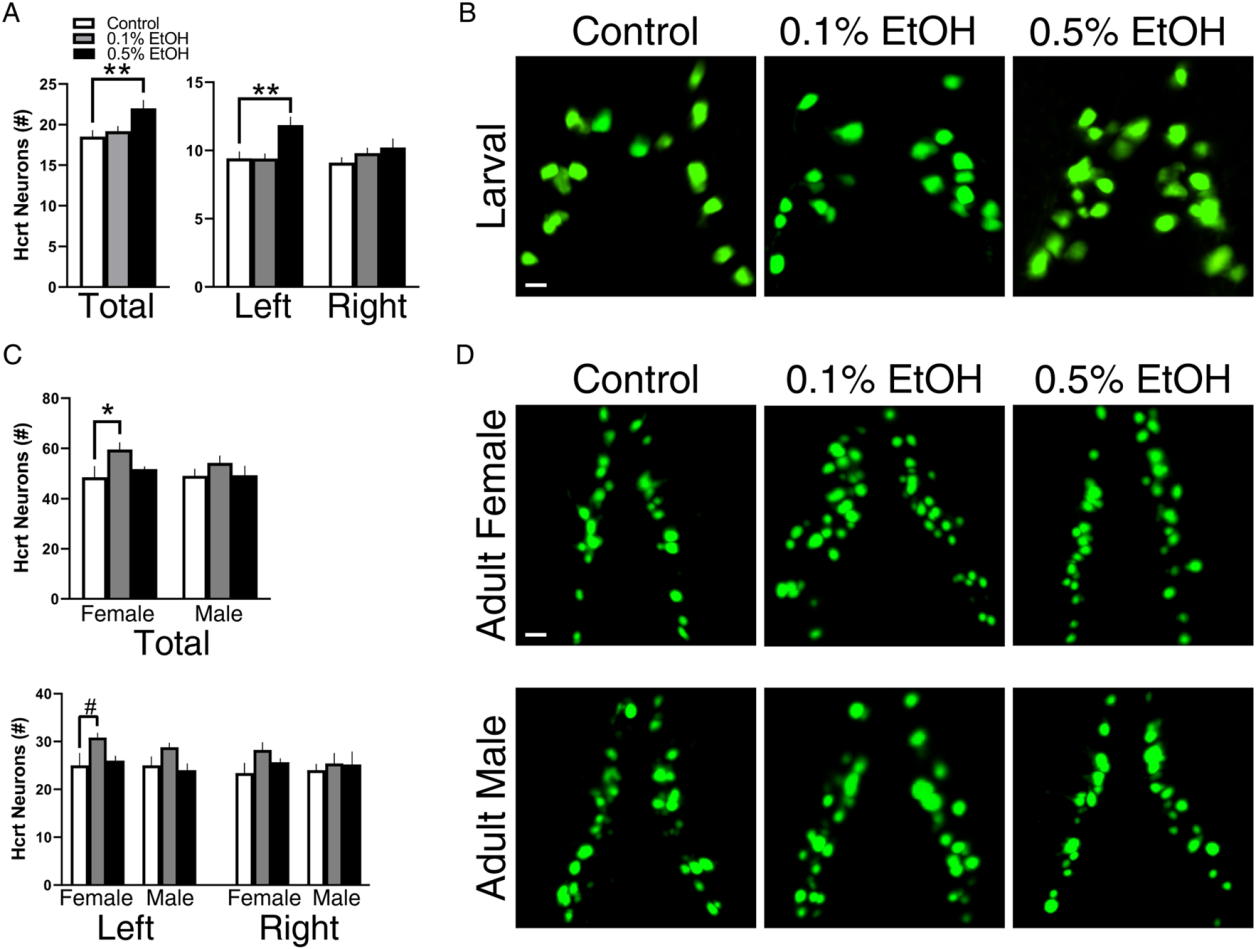
Effects of embryonic EtOH from 22 to 24 hpf on Hcrt neuron number in the AH measured in 6 dpf larval zebrafish using live-imaging and in adult zebrafish using iDISCO clearing. (**A**) EtOH at 0.5% (n = 14) but not 0.1% (n = 20) increases the total number of Hcrt neurons and the number of Hcrt neurons on the left but not right side in 6 dpf larval zebrafish compared to control (n = 19). (**B**) These effects in larval zebrafish are illustrated in representative confocal images of the AH. They show that 0.5% but not 0.1% increases the total and left side number of GFP-positive Hcrt neurons (green) in 6 dpf larval zebrafish compared to control. (**C**) EtOH in adult females at 0.1% (n = 5) but not 0.5% (n = 6) increases the total number of Hcrt neurons and increases the number of Hcrt neurons on the left but not right side of the AH compared to control (n = 4), with no changes occurring in males at 0.1% (n = 5) or 0.5% (n = 5) relative to control (n = 5). (**D**) These effects in adults are illustrated in representative confocal images showing the total population of Hcrt neurons in the AH. They show that 0.1% but not 0.5% increases the total number of Hcrt neurons in females (middle row) but not males (bottom row) and increases the number of Hcrt neurons on the left but not right side only in adult females compared to control. Results are shown as means ± standard errors. *p < 0.05, **p < 0.01. ^#^p < 0.1.

Based on these asymmetric effects of EtOH on the proliferation of Hcrt neurons shown with analysis of the total population using IF in larval zebrafish, we sought to confirm these findings using live imaging in larvae and also determine using iDISCO clearing in female and male adult zebrafish whether these effects are long-lasting and possibly sexually dimorphic. Similar to the above experiment using IF, live imaging at 6 dpf showed that EtOH increased the number of Hcrt neurons (*F*(2, 50) = 4,616, *p* = 0.0145), specifically at the 0.5% concentration (*p* = 0.0100) (Fig. 3A), as shown in the photomicrographs (Fig. 3B). This EtOH-induced increase in Hcrt was long lasting and evident in adult fish. While there was no interaction between embryonic EtOH and sex, the ANOVA revealed a significant main effect of embryonic EtOH on total Hcrt neuron number (*F*(2, 24) = 3.719, *p* = 0.0392), and the post-hoc analysis showed after 0.1% EtOH a significant increase in Hcrt neurons in females (*p* = 0.0460) that was not evident in males (*p* = 0.4033, ns) (Fig. 3C), as shown in the photomicrographs (Fig. 3D).

We next evaluated the asymmetry of Hcrt neurons. In larvae, the ANOVA revealed a significant interaction between embryonic EtOH and brain side (*F*(2, 50) = 3.400, *p* = 0.0413), with a significant main effect of embryonic EtOH (*F*(2, 50) = 4.846, *p* = 0.0119), and the post-hoc analysis showed that 0.5% EtOH compared to control significantly increased the total number of Hcrt neurons on the left (*p* = 0.0094) but not right (p = 0.2485, ns) side of the AH (Fig. 3A), as shown in the photomicrographs (Fig. 3B). This asymmetry was similarly evident in adults. While showing no interaction between EtOH, brain side, and sex, the ANOVA revealed a significant main effect of embryonic EtOH (*F*(2, 24) = 3.719, *p* = 0.0392), and the post-hoc analysis again showed after 0.1% EtOH an increase in Hcrt neurons that approached significance on the left (*p* = 0.0655, ns) but not right (*p* = 0.1159, ns) side of the AH in females with no effects occurring in males (Fig. 3C), as shown in the photomicrographs (Fig. 3D).

### Embryonic EtOH exposure decreases locomotor activity in larvae and in female but not male adults, only under novel conditions

**Figure 4:**
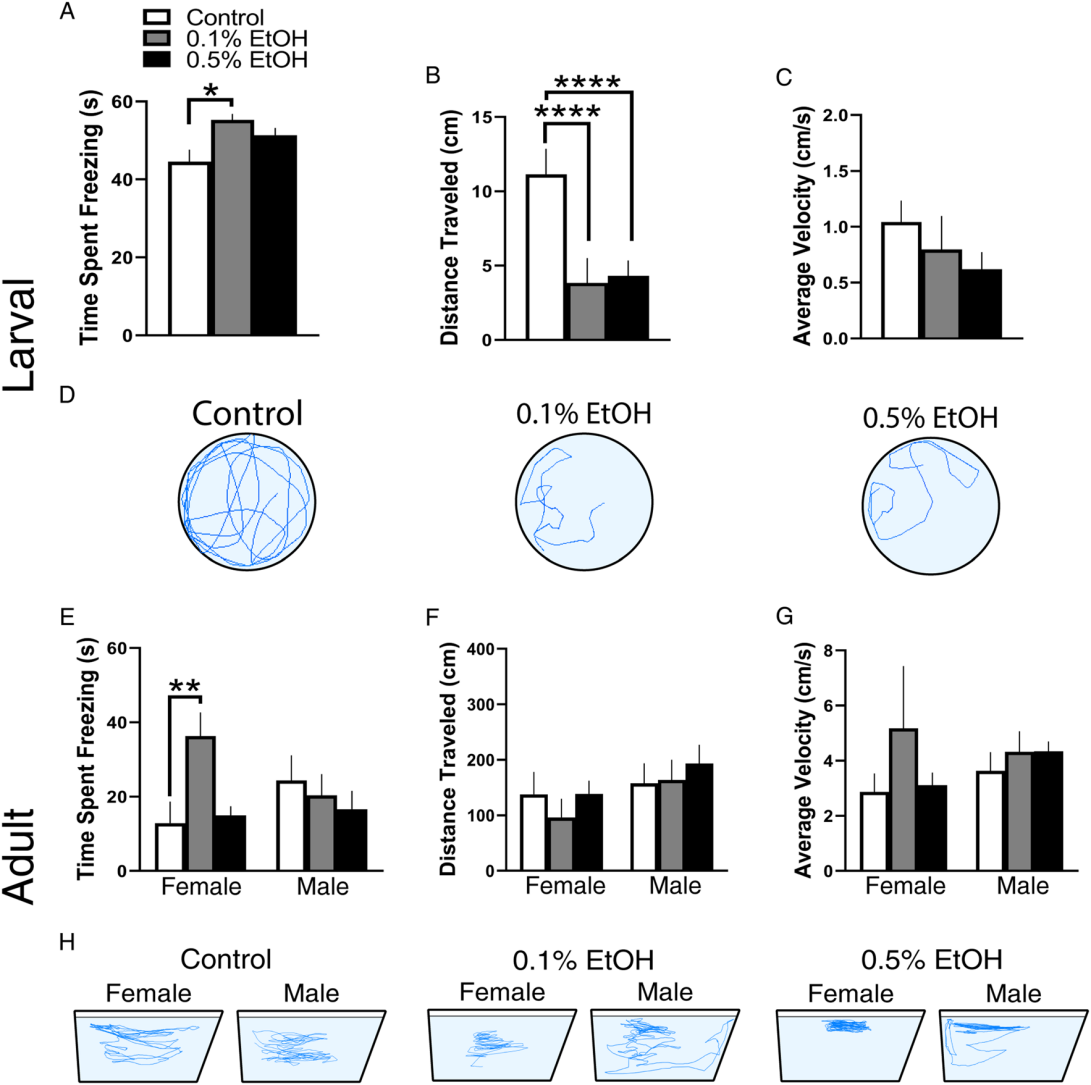
Effects of embryonic EtOH from 22 to 24 hpf on locomotor activity measured in 6 dpf larval and adult zebrafish under novel conditions (min 1) (**A**) EtOH at 0.1% (n = 19) increases time spent freezing in larval zebrafish compared to control (n = 24), with no change in the 0.5% group (n = 30). (**B**) EtOH at 0.1% and 0.5% decreases the distance traveled in larval zebrafish compared to control. (**C**) EtOH at 0.1% and 0.5% has no effect on the average velocity in larval zebrafish compared to control. (**D**) Visual tracking representation of the open-field test as viewed from above showing that embryonic EtOH decreased locomotor activity in larval zebrafish as compared to control. (**E**) EtOH in adult females at 0.1% (n = 8) but not 0.5% (n = 10) increases in min 1 the time spent freezing compared to the control (n = 9), with no changes occurring in adult males at 0.1% (n = 10) or 0.5% (n = 7) relative to control (n = 8). (**F**) EtOH at 0.1% and 0.5% has no effect on the distance traveled in both male and female adult zebrafish compared to control. (**G**) EtOH at 0.1% and 0.5% has no effect in min 1 on the average velocity in both male and female adult zebrafish. (**H**) Visual tracking representation as seen from the side in the novel tank test. Results are shown as means ± standard errors. *p < 0.05, **p < 0.01, ****p < 0.0001.

To examine the behavioral effects of embryonic EtOH early in development in 6 dpf larvae, we measured locomotor activity behaviors in an open-field arena, under novel conditions (min 1) and after habituation (min 10). In the analysis of locomotor activity, while revealing no significant interactions for the measures of time spent freezing or average velocity, the ANOVA revealed main effects of time on time spent freezing (*F*(1, 70) = 17.89, *p* < 0.0001) and velocity (*F*(1, 70) = 24.09, *p* < 0.0001) and a significant interaction between EtOH and time for the measure of distance traveled (*F*(2, 70) = 5.037, *p* = 0.0090), with significant main effects of both EtOH (*F*(2, 70) = 6.388, *p* = 0.0028) and time (*F*(1, 70) = 5.175, *p* = 0.0260). For all larval measures, the post-hoc analyses of locomotor activity showed that embryonic EtOH produced changes only under novel conditions during min 1, with no effects occurring after habituation in min 10 (see in Supplementary Table, S1). In the first min of testing, there was an increase in time spent freezing by the 0.1% group (*p* = 0.0145) (Fig. 4A), a decrease in distance traveled by the 0.1% group (*p* < 0.0001) and 0.5% group *(p* < 0.0001) relative to control (Fig. 4B), with no changes in velocity (Fig. 4C). These results show that the behavioral effects of EtOH occurred only under novel conditions, with freezing behavior significantly increased and distance traveled decreased in larvae as shown by representative swim paths (Fig. 4D).

To determine in adults if these behavioral effects are long-lasting and sexually dimorphic, we measured locomotor activity in adult male and female zebrafish under novel conditions and after habituation. As with larvae, EtOH at 0.1% inhibited locomotor activity specifically under novel conditions at the start of the test session. While showing no significant interactions for distance traveled or average velocity, the ANOVA revealed a significant three-way interaction between embryonic EtOH, time, and sex, for the measure of time spent freezing (*F*(2,46) = 4.595, *p* = 0.0152), with significant main effects of embryonic EtOH (*F*(2,46) = 3.991, *p* = 0.0252) and time (*F*(1,46) = 27.49, *p* < 0.0001). As in larvae, adult females in the 0.1% condition spent more time freezing during the first min compared to control females (*p* = 0.0046) (Fig. 4E), with no effect on freezing evident in 0.1% males (*p* = 0.6307, ns). There were no changes in distance traveled (Fig. 4F) or velocity (Fig. 4G) in males or females. These results are illustrated by representative swim paths (Fig. 4H). In contrast to these changes at 1 min, there were no effects of EtOH on locomotor activity at min 10 in either female or males adults (see Supplementary Table S1).

### Embryonic EtOH exposure produces sexually dimorphic changes in voluntary EtOH consumption, anxiety-like behavior and aggressio﻿n

**Figure 5:**
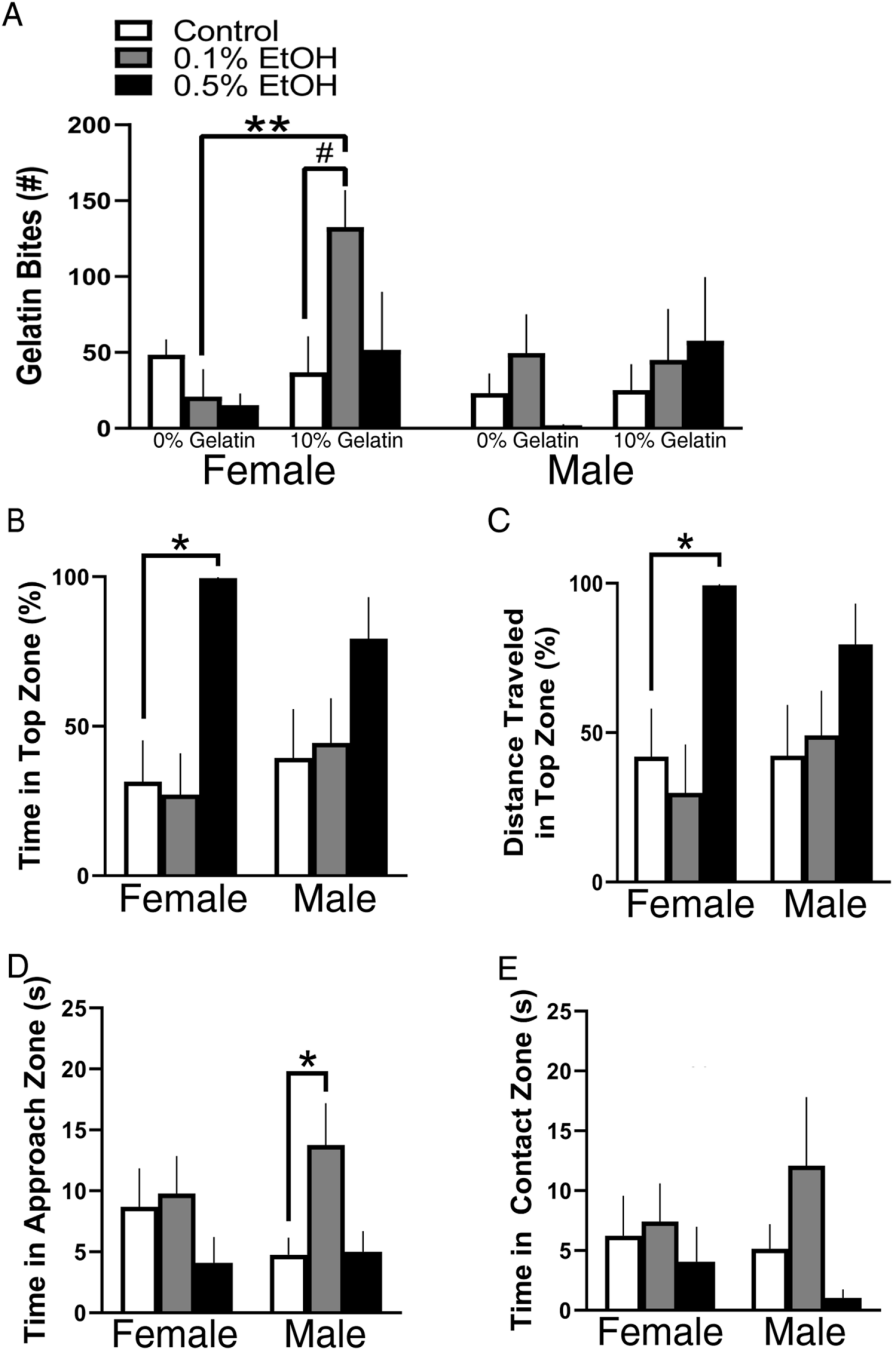
Effects of embryonic EtOH from 22 to 24 hpf on voluntary EtOH consumption, as well as anxiety-like and aggressive behaviors measured in adult female and male zebrafish under novel conditions (min 1). (**A**) EtOH in females at 0.1% (n = 7), but not 0.5% (n = 7), increases consumption of 10% EtOH gelatin relative to the amount of 0% EtOH gelatin consumed by 0.1% females. EtOH at 0.1% (n = 7) also produces a near significant increase in consumption of 10% EtOH gelatin relative to the amount of 10% gelatin consumed in the control group (n = 10), with no changes occurring in males at 0.1% (n = 8) or 0.5% (n = 7) relative to control (n = 9). (**B**) EtOH in females at 0.5% (n = 9) but not 0.1% (n = 7) increases the percent of time traveled in the top zone compared to control (n = 7), with no changes occurring in males at 0.1% (n = 9) or 0.5% (n = 7) relative to control (n = 8) males. (**C**) EtOH in females at 0.5% but not 0.1% increases the percent distance traveled in the top zone compared to control, with no changes occurring in males at 0.1% or 0.5% relative to control. (**D**) EtOH in males at 0.1% (n = 10) but not 0.5% (n = 7) increases the time spent in the approach zone compared to control (n = 9) during min 1, with no changes occurring at min 10 or in females at 0.1% (n = 8) or 0.5% (n = 10) relative to control (n = 9), indicating an increase in aggression only in males. (**E**) EtOH had no effect on the time spent in the contact zone in both males at 0.1% or 0.5% compared to control, nor in females at 0.1% or 0.5% compared to control. Results are shown as means ± standard errors. *p < 0.05, **p < 0.01.

We next investigated the effects of embryonic EtOH exposure on voluntary EtOH consumption, anxiety-like behaviors and aggression in female and male adult zebrafish. In the analysis of EtOH consumption, the ANOVA revealed a significant main effect of gelatin conditions (*F*(1, 35) = 4.666, *p* = 0.0377), with no significant interactions. Post-hoc analysis showed that the females in response to 0.1% EtOH exposure exhibited the greatest consumption of 10% gelatin, consuming significantly more of the 10% EtOH gelatin compared to control gelatin (*p* = 0.0074), and their consumption of 10% gelatin tended to be greater than that of the control group (*p* = 0.0745, ns) (Fig. 5A). Analysis of male zebrafish, in contrast, showed no significant changes in EtOH consumption, demonstrating this EtOH-induced behavior to be strongly sex dependent. We next measured if embryonic EtOH affected exploration within the novel tank test to evaluate anxiety-like behavior and found a decrease in these behaviors under novel conditions only in 0.5% females during min 1 but not min 10. The ANOVA revealed significant interactions between embryonic EtOH and time for percent distance traveled in the top zone (*F*(2,40) = 4.294, *p* = 0.0204) and percent time spent in the top zone (*F*(2,40) = 4.787, *p* = 0.0137) and significant main effects of EtOH (*F*(2,46) = 7.140, *p* = 0.0020) and time (*F*(1,40) = 6.000, *p* = 0.0188) for time spent in the top zone and of EtOH (*F*(2,46) = 6.593, *p* = 0.0030) and time (*F*(1,40) = 6.469, *p* = 0.0149) for distance traveled in the top zone. Post-hoc analysis revealed at the 0.5% concentration only in females a significant increase in the percentage of time (*p* = 0.0282) (Fig. 5B) and distance traveled in the top zone (*p* = 0.0207) (Fig. 5C) at min 1 relative to control, with no changes occurring at min 10 (see Supplementary Table S1). Lastly, we sought to determine if embryonic EtOH produced changes in aggressive behaviors in adult fish. While showing no significant interactions between embryonic EtOH, sex, and time, there was a significant main effect of time on time in approach zone (*F*(1, 47) = 27.03, *p* < 0.0001) and time in contact zone (*F*(1, 47) = 13.64, *p* = 0.0006). Post-hoc analysis revealed at the 0.1% concentration relative to control an increase in time spent in the approach zone in males (*p* = 0.0331) but not in females (*p* = 0.7927, ns) (Fig. 5D) with no change in time in the contact zone (Fig. 5E), demonstrating a sexually dimorphic, EtOH-induced increase in aggressive behaviors.

## Discussion

We demonstrate here that embryonic exposure to EtOH, administered at low-moderate concentrations during the period of peak hypothalamic neuronal development, stimulates cell proliferation throughout the entire AH but increases asymmetrically the proliferation specifically of Hcrt neurons only on the left side of the AH. This EtOH-induced stimulatory effect on Hcrt neurons is accompanied by an increase in freezing behavior specifically under novel conditions, with these effects observed in both larvae and adults indicating that they are long lasting. Further, analyses of adults show these neuronal and behavioral effects to be strongly sex dependent, with an increase in voluntary EtOH consumption and freezing behavior along with the asymmetric increase in Hcrt neurons evident only in females and an increase in aggressive behavior observed only in males without any change in Hcrt neurons. Overall, these long-lasting and sexually dimorphic effects are comparable to those observed in rodents, demonstrating the validity and strengths of the zebrafish model in studying the neurobehavioral effects of embryonic EtOH exposure.

Using two-dimensional image analysis, we have previously demonstrated that embryonic EtOH exposure in zebrafish, similar to prenatal exposure in rats^9^, increases the number of differentiated neurons throughout the developing hypothalamus^16^ and of neurons positive for the proliferation marker BrdU in the AH^11^, supporting the idea that low-moderate EtOH concentrations stimulate hypothalamic neurogenesis. In the present study, we utilized volume imaging and three-dimensional quantification of cell proliferation and Hcrt neuron number, methods uniquely suited to zebrafish that allowed for a more detailed quantitative analysis and to precisely count the absolute number of proliferating cells and Hcrt neurons throughout the whole AH. This volume imaging of both sides of the brain, an analysis difficult to perform accurately when using tissue sections, revealed clear EtOH-induced asymmetries in the Hcrt system in larvae that persisted into adulthood. Specifically, while asymmetrically increasing Hcrt neuronal number as described^31^, EtOH increased the number of Hcrt neurons positive for the proliferation marker EdU only on the left side of the brain, and the number of Hcrt neurons was strongly positively correlated with the number of EdU-positive DAPI cells, again only on the left. These results suggest that the asymmetric increase in Hcrt neuronal number derives specifically from an asymmetric increase in Hcrt neurogenesis, rather than a region-wide stimulation of cell proliferation that occurred throughout both sides of the AH. It may also result from an increase in differentiation of neuronal progenitor cells into Hcrt neurons, with prior evidence showing prenatal EtOH exposure to increase the density in rats of radial glia progenitor cells in the neuroepithelium^32^, and EtOH in vitro to alter the cell fate of neuronal stem and progenitor cells^50,51^.

While little is known about the asymmetric properties of neurons in the hypothalamus and whether EtOH has differential effects on both sides of the brain, there is evidence in humans that the asymmetry itself may be a contributing factor to the behavioral disturbances, with in utero EtOH exposure that stimulates the consumption of EtOH found to have asymmetric effects on the size of different brain regions^52^ and the risk for alcohol use disorder shown to be increased in adolescents with asymmetrical brain morphology^33^. A possible mechanism underlying the asymmetric nature of this EtOH-induced effect on Hcrt neurons may be the neuroimmune system, such as the chemokine receptor Cxcr4b. This receptor is found to stimulate neurogenesis in zebrafish as it first occurs on the left side of the habenula^53^ and to be stimulated by embryonic EtOH exposure in hypothalamic neurons in zebrafish^16^ and specifically in Hcrt neurons in rats^32^. Our finding here that this asymmetric EtOH-induced increase in Hcrt neurons occurs similarly in adults as in larvae demonstrates that this effect observed shortly after embryonic EtOH exposure is long lasting. This is consistent with other studies showing effects in the larval brain to persist into adulthood, including an EtOH-induced increase in proliferation of hypothalamic neurons^11^ and reduction in whole brain measures of monoamines, glutamate and voltage-gated ion channels^54–58^.

Novel environments represent a potential threat to animals and induce characteristic behavioral responses, including freezing behavior, which act to minimize detection by potential predators. Our new finding, that the low concentration of 0.1% EtOH produces a long lasting increase in freezing behavior only under novel conditions and not after habituation to the environment, suggests that only the immediate response to the novel environment is affected by embryonic EtOH exposure and that these novelty-induced behaviors may reflect an elevation in anxiety or fear^59^. Although studies of locomotor activity^35,36^ typically fail to distinguish between behavioral responses to novelty and behaviors after habituation and generally average behavior over the entire testing session, our results here in both larvae and adult fish are consistent with a study in adolescent rats showing prenatal EtOH exposure to reduce horizontal locomotion and rearing behavior in response to novelty but not after habituation^60^. Our additional findings in the novel tank test, showing that EtOH consistent with prior reports^11,61^ reduces anxiety-like behaviors under novel conditions only at the 0.5% but not 0.1% concentration while increasing Hcrt neurons only at the 0.1% concentration, suggests that the reduced anxiety at the 0.5% dose in adult females may be attributed to a mechanism other than Hcrt, such as a reduced release of cortisol that is also observed at higher EtOH concentrations in zebrafish^62^.

Our behavioral analyses in adult zebrafish embryonically exposed to EtOH revealed marked changes in behavior that were strongly sexually dimorphic. The increase in both voluntary EtOH consumption and freezing behavior induced by EtOH exposure at the 0.1% concentration occurred only in females, while the increase in aggressive behavior produced by 0.1% EtOH was observed only in males. Further, control females exhibited a trend towards higher aggression compared to control males, a finding consistent with prior reports in zebrafish^63^. The increase in EtOH consumption in adult females as described in juvenile zebrafish^11^ agrees with other reports in rats showing prenatal EtOH exposure to increase alcohol intake and preference in females more than males^35,40,64^. It is also consistent with clinical findings that females exposed in utero to low-moderate concentrations of EtOH are more susceptible than males to engage in problem drinking^65^ and exhibit an increase in alcohol drinking during adolescence^8^ and adulthood^66^. Our finding in males, that embryonic EtOH exposure increases aggressive behavior at the 0.1% but not 0.5% concentration, agrees with studies describing this same result in rodents^67^ and clinical evidence showing that young boys prenatally exposed to EtOH at the low but not high levels exhibit more externalizing behaviors than girls^6^.

The long lasting effects of EtOH exposure on the Hcrt system produced by the lower 0.1% concentration was also found in adult fish to be sex dependent as shown in rats^32^, with the asymmetric increase in the proliferation and number of Hcrt neurons occurring in females but not in males. Although not tested here directly, the possibility that this increase in Hcrt neurons is causally related to the sexually dimorphic and long lasting behavioral effects in females is supported by studies showing Hcrt administration to promote alcohol consumption in zebrafish^11^ and rodents^68^, Hcrt mRNA to be positively correlated with an increase in freezing behavior in rodents^69^, and elevated Hcrt levels in cerebrospinal fluid to be linked to anxiety disorders^70^, which are commonly comorbid with alcohol use disorder^71^. Further studies in zebrafish, testing if these EtOH induced behavioral alterations are observed in Hcrt knockout lines or diminished after inducible laser ablation of specific Hcrt neurons affected by EtOH exposure, are needed to provide direct evidence for a causal relationship between the EtOH induced increase in Hcrt neurogenesis and the alterations in behavior.

Although the precise molecular mechanisms underlying the sexually dimorphic effects of EtOH on Hcrt neurons are yet to be characterized, our evidence showing EtOH to stimulate both the chemokine receptor Cxcr4 along with Hcrt neuron density in females more than males^32^ indicates that this chemokine system may be involved in these effects. With other evidence showing EtOH to stimulate estrogen receptor α (Erα) expression^72^, it is also possible that EtOH upregulates Hcrt neurons more strongly in females through the activation of the Erα, although there is no published evidence showing Erα to be expressed on Hcrt neurons. A possible involvement of Erα in this asymmetric increase in Hcrt neurons observed only in females is further supported by evidence that this receptor, which is well conserved in zebrafish^73^, stimulates neural stem cell proliferation and differentiation into neurons^74,75^, is expressed on the cell surface of embryonic hypothalamic neurons^76^, and is asymmetrically affected by EtOH exposure in female rats^77^. In contrast to females, we observed an increase in aggressive behavior in males in the absence of changes in Hcrt neuron number, indicating that this effect may not be dependent on stimulation of Hcrt neurogenesis. There is clinical evidence, however, indicating that males homozygous for a variant in the Hcrt receptor 1 (*hcrtr1*) exhibit an increase in aggressive behaviors^78^, suggesting that alterations in Hcrt signaling may be involved in driving these behaviors in males. Further studies in zebrafish are needed to test this possibility.

In summary, this study demonstrates that embryonic EtOH administration, at a low-moderate concentration and for only 2 h during peak hypothalamic development, produces changes in neuronal development and behavior that are long lasting and sexually dimorphic. It causes an asymmetric increase in the proliferation and number of Hcrt neurons along with an increase in voluntary EtOH consumption and novelty-induced freezing behavior only in females, suggesting that the asymmetric change in Hcrt neurons may be causally related to these behavioral effects. In males, however, it causes an increase in aggressive behavior which while not associated with an increase in Hcrt neurons may have some relation to an EtOH-induced change in the signaling of Hcrt neurons. Notably, these sex-dependent effects in zebrafish are in agreement with rodent and clinical studies, indicating that the effects of EtOH on the embryo are well conserved across species and the zebrafish is an advantageous model for investigating the neuronal changes underlying disturbances in behavior.

## Supplementary Information

Supplementary Information.
