# Supplementary material for: Sexually dimorphic and asymmetric effects of embryonic ethanol exposure on hypocretin/orexin neurons as related to behavioral changes in zebrafish
Source: Sci Rep. 2021 Aug 9;11:16078. doi: 10.1038/s41598-021-95707-y (PMC8352948; doi:10.1038/s41598-021-95707-y)
Supplement: Supplementary file 1 — Supplementary Information. [file 41598_2021_95707_MOESM1_ESM.pdf]

SUPPLEMENTARY INFORMATION

Sexually dimorphic and asymmetric effects of embryonic ethanol exposure on hypocretin/orexin neurons as related to behavioral changes in zebrafish

Adam D. Collier[1], Nushrat Yasmin[1], Nailya Khalizova[1], Samantha Campbell[1], Amanda Onoichenco[1], Milisia Fam[1], Avi S. Albeg[1], Sarah F. Leibowitz[1*]

[1]Laboratory of Behavioral Neurobiology, The Rockefeller University, New York, NY

*Address for Correspondence:

Sarah F. Leibowitz,

Laboratory of Behavioral Neurobiology,

The Rockefeller University,

1230 York Avenue, New York, NY 10065, USA.

Phone: 212-327-8378, Fax: 212-327-8447,

E-mail: leibow@rockefeller.edu

Supplementary Table S1. Effects of embryonic EtOH from 22-24 hpf on locomotor activity in 6 dpf larval and adult zebrafish, and on anxiety-like behavior and aggression in adult zebrafish measured at minute 10.

**Locomotor Activity:**	**Embryonic EtOH**	**Larval**	**Adult**	
			**Female**	**Male**
Time Spent Freezing (s)	Control	41.226 ± 3.118	8.659 ± 3.850	7.336 ± 2.885
	0.1% EtOH	43.791 ± 3.099	9.984 ± 2.972	14.282 ± 3.903
	0.5% EtOH	43.176 ± 2.520	6.978 ± 1.743	5.504 ± 1.767
Distance Traveled (cm)	Control	5.295 ± 1.074	216.668 ± 38.789	275.990 ± 44.688
	0.1% EtOH	4.255 ± 1.044	253.786 ± 48.497	206.381 ± 39.985
	0.5% EtOH	3.837 ± 0.712	274.540 ± 23.598	302.068 ± 49.430
Velocity (cm/s)	Control	0.200 ± 0.031	4.196 ± 0.614	5.022 ± 0.648
	0.1% EtOH	0.204 ± 0.039	4.826 ± 0.723	4.177 ± 0.537
	0.5% EtOH	0.195 ± 0.039	5.125 ± 0.328	5.451 ± 0.753
**Anxiety:**				
Time Traveled in Top Zone (%)	Control		71.093 ± 10.537	63.373 ± 12.160
	0.1% EtOH		56.911 ± 11.963	63.345 ± 11.668
	0.5% EtOH		79.052 ± 6.745	80.612 ± 9.345
Distance Traveled in Top Zone (%)	Control		73.347 ± 8.415	65.152 ± 12.147
	0.1% EtOH		58.164 ± 11.557	66.732 ± 11.013
	0.5% EtOH		82.042 ± 5.765	82.343 ± 8.785
Top Zone Entries (#)	Control		3.667 ± 0.898	3.875 ± 0.811
	0.1% EtOH		6.375 ± 1.603	4.300 ± 1.342
	0.5% EtOH		5.600 ± 1.572	4.286 ± 0.993
**Aggression:**				
Time in Contact Zone (s)	Control		17.313 ± 3.990	10.499 ± 3.750

	0.1% EtOH		17.520 ± 4.350	12.421 ± 3.662
	0.5% EtOH		14.548 ± 4.236	7.011 ± 3.727
Time in Approach Zone (s)	Control		19.672 ± 4.043	15.631 ± 3.908
	0.1% EtOH		20.676 ± 4.194	14.104 ± 3.221
	0.5% EtOH		16.344 ± 2.908	11.494 ± 3.687

Data are represented as mean ± SEM.

Supplementary Table S2. 2-Way ANOVA results for cell proliferation and neurogenesis in AH of 6 dpf larval zebrafish following embryonic ethanol.

**Main Effects:**	**Embryonic EtOH**			**Side**		
	df	**F**	***p***	***df***	**F**	***p***
DAPI+ Cells in AH (#)	2,14	0.2477	0.7840	**1,14**	**8.253**	**0.0123***
EdU+ Cells in AH (#)	2,14	1.766	0.2070	**1,14**	**4.630**	**0.0494***
EdU+/DAPI+ Cells in AH (%)	**2,14**	**4.961**	**0.0235***	1,14	0.03445	0.8554
Hcrt Neurons (#)	**2,16**	**6.885**	**0.0070****	1,16	0.2545	0.6208
EdU+/Hcrt+ Neurons (#)	**2,17**	**5.657**	**0.0131***	1,17	4.311	0.0534

**Two-Way Interactions:**	**Embryonic EtOH x Side**		
	df	**F**	***p***
DAPI+ Cells in AH (#)	2,14	1.260	0.3138
EdU+ Cells in AH (#)	2,14	0.7343	0.4974
EdU+/DAPI+ Cells in AH (%)	2,14	0.03445	0.9662
Hcrt Neurons (#)	2,16	0.08241	0.9213
EdU+/Hcrt+ Neurons (#)	**2,17**	**4.588**	**0.0255***

*Significant effects are boldface. *p<0.05; **p<0.01.*

Supplementary Table S3. Pearson correlation between measures of cell proliferation and number of Hcrt neurons in the AH of 6 dpf larval zebrafish following embryonic ethanol.

**Correlations:**	**Total Hcrt+**			**Left Hcrt+**			**Right Hcrt+**	
	**r**	***p***		**r**	***p***		**r**	***p***
Total DAPI+	-0.190	0.480	Left DAPI+	0.051	0.853	Right DAPI+	-0.123	0.639
Total EdU+	0.347	0.188	Left EdU+	0.460	0.073	Right EdU+	0.083	0.750
Total EdU+/ DAPI+	**0.690**	**0.003****	Left EdU+/ DAPI+	**0.594**	**0.015***	Right EdU+/ DAPI+	0.269	0.296
Total EdU+/ Hcrt+	**0.514**	**0.041***	Left EdU+/ Hcrt+	0.431	0.096	Right EdU+/ Hcrt+	0.171	0.513

*Significant effects are boldface. *p<0.05; **p<0.01*

Supplementary Table S4. 2- Way ANOVA results for Hcrt neuron count in live imaging of AH in 6 dpf larval zebrafish following embryonic ethanol.

**Main Effects:**	**Embryonic EtOH**			**Side**		
	df	**F**	***p***	***df***	**F**	***p***
Hcrt Neurons (#)	**2,50**	**4.846**	**0.0119***	1,50	2.746	0.1038
**Two-Way Interactions:**	**Embryonic EtOH x Side**					
	df	**F**	***p***			
Hcrt Neurons (#)	**2,50**	**3.400**	**0.0413***			

*Significant effects are boldface. *p<0.05*

Supplementary Table S5. 3-Way ANOVA results for Hcrt neurons count in iDISCO analysis of the AH in adult zebrafish following embryonic ethanol.

**Main Effects:**	**Embryonic EtOH**			**Side**			**Sex**		
	df	**F**	***p***	***df***	**F**	***p***	***df***	**F**	***p***
Hcrt Neurons (#)	**2,24**	**3.719**	**0.0392***	1,24	2.448	0.1308	1,24	0.9203	0.3469
**Two-Way Interactions:**	**Embryonic EtOH x Side**			**Embryonic EtOH x Sex**			**Side x Sex**		
	df	**F**	***p***	***df***	**F**	***p***	***df***	**F**	***p***
Hcrt Neurons (#)	2,24	2.124	0.1415	2,24	0.4229	0.6599	1,24	0.0000657 1	0.9936

Three-Way Interactions:	Embryonic EtOH x Side x Sex		
	df	**F**	***p***
Hcrt Neurons (#)	2,24	0.3391	0.7158

*Significant effects are boldface. *p<0.05*

Supplementary Table S6. 2-Way ANOVA results for behavioral assay of locomotor activity in 6 dpf larval zebrafish following embryonic ethanol.

Main Effects:	Embryonic EtOH			Time		
	df	**F**	***p***	***df***	**F**	***p***
Time Spent Freezing (s)	2,70	2.362	0.1017	**1,70**	**17.89**	**<0.0001********
Distance Traveled (cm)	2,70	**6.388**	**0.0028****	**1,70**	**5.175**	**0.0260***
Average Velocity (cm/s)	2,70	1.202	0.3068	**1,70**	**24.09**	**<0.0001********

Two-Way Interactions:	Embryonic EtOH x Time		
	df	**F**	***p***
Time Spent Freezing (s)	2,70	1.577	0.2139
Distance Traveled (cm)	**2,70**	**5.037**	**0.0090****
Average Velocity (cm/s)	2,70	1.037	0.3597

*Significant effects are boldface. *p<0.05; **p<0.01; ****p<0.0001*

Supplementary Table S7. 3-Way ANOVA results for behavioral assays of locomotor activity, anxiety-like behavior, and aggression in adult zebrafish following embryonic ethanol.

Main Effects:	Embryonic EtOH			Time			Sex		
	df	**F**	***p***	***df***	**F**	***p***	***df***	**F**	***p***
Time Spent Freezing (s)	**2,46**	**3.991**	**0.0252***	**1,46**	**27.49**	**<0.0001 ********	1,46	0.006254	0.9373
Distance Traveled (cm)	2,46	0.9889	0.3797	**1,46**	**61.43**	**<0.0001 ********	1,46	1.200	0.2790
Average Velocity (cm/s)	2,46	0.6813	0.5110	1,46	3.925	0.0536	1,46	0.2745	0.6029

Time Traveled in Top Zone (%)	**2,46**	**7.140**	**0.0020 ****	**1,40**	**6.000**	**0.0188***	1,46	0.002693	0.9588
Distance Traveled in Top Zone (%)	**2,46**	**6.593**	**0.0030 ****	**1,40**	**6.469**	**0.0149***	1,46	0.0003714	0.9847
Top Zone Entries (#)	2,46	0.6954	0.5041	**1,46**	**37.78**	**<0.0001 ******	1,46	0.6686	0.4177
Time in Contact Zone (s)	2,47	1.933	0.1560	**1,47**	**13.64**	**0.0006 *****	1,47	1.820	0.1838
Time in Approach Zone (s)	2,47	2.240	0.1177	**1,47**	**27.03**	**<0.0001 ******	1,47	1.394	0.2437

**Two-Way Interactions:**	**Embryonic EtOH x Time**			**Embryonic EtOH x Sex**			**Time x Sex**		
	df	**F**	***p***	***df***	**F**	***p***	***df***	**F**	***p***
Time Spent Freezing (s)	2,46	0.8102	0.4510	2,46	1.299	0.2828	1,46	0.09702	0.7568
Distance Traveled (cm)	2,46	0.3086	0.7360	2,46	0.1347	0.8743	1,46	1.583	0.2147
Average Velocity (cm/s)	2,46	1.647	0.2038	2,46	0.9850	0.3812	1,46	0.05533	0.8151
Time Traveled in Top Zone (%)	**2,40**	**4.787**	**0.0137***	2,46	0.4451	0.6435	1,40	0.04291	0.8369
Distance Traveled in Top Zone (%)	**2,40**	**4.294**	**0.0204***	2,46	0.5342	0.5897	1,40	0.02959	0.8643
Top Zone Entries (#)	2,46	0.9024	0.4127	2,46	0.1748	0.8402	1,46	1.156	0.2879
Time in Contact Zone (s)	2,47	0.2899	0.7497	2,47	0.4336	0.6507	1,47	2.625	0.1119
Time in Approach Zone (s)	2,47	0.9186	0.4061	2,47	0.1614	0.8515	1,47	2.723	0.1056

**Three-Way Interactions:**	**Embryonic EtOH x Time x Sex**		
	df	**F**	***p***
Time Spent Freezing (s)	**2,46**	**4.595**	**0.0152***
Distance Traveled (cm)	2,46	2.745	0.0748
Average Velocity (cm/s)	2,46	0.1467	0.8639

Time Traveled in Top Zone (%)	2,40	0.8955	0.4164
Distance Traveled in Top Zone (%)	2,40	0.8726	0.4257
Top Zone Entries (#)	2,46	1.025	0.3670
Time in Contact Zone (s)	2,47	0.1935	0.8247
Time in Approach Zone (s)	2,47	0.8527	0.4327

*Significant effects are boldface. *p<0.05; **p<0.01; ***p<0.001; ****p<0.0001*

Supplementary Table S8. 3-Way ANOVA results for gelatin intake by adult zebrafish following embryonic ethanol.

**Main Effects:**	**Embryonic EtOH**			**Gelatin**			**Sex**		
	df	**F**	***p***	***df***	**F**	***p***	***df***	**F**	***p***
Gelatin Intake	2,35	1.865	0.1699	**1,35**	**4.666**	**0.0377***	1,35	1.374	0.2491
**Two-Way Interactions:**	**Embryonic EtOH x Gelatin**			**Embryonic EtOH x Sex**			**Gelatin x Sex**		
	df	**F**	***p***	***df***	**F**	***p***	***df***	**F**	***p***
Gelatin Intake	2,35	1.546	0.2273	2,35	0.2671	0.7671	1,35	0.9032	0.3484
**Three-Way Interactions:**	**Embryonic EtOH x Gelatin x Sex**								
	df	**F**	***p***						
Gelatin Intake	2,35	2.361	0.1091						

*Significant effects are boldface. *p<0.05*
